# Ethylene-Vinyl Acetate Copolymer as a Polyfunctional Modifier for Low-Viscosity Photosensitive Compositions

**DOI:** 10.3390/polym17202787

**Published:** 2025-10-17

**Authors:** Dmitriy A. Bazhanov, Uliana V. Nikulova, Ramil R. Khasbiullin, Nikita Yu. Budylin, Elizaveta V. Ermakova, Aleksey V. Shapagin

**Affiliations:** Frumkin Institute of Physical Chemistry and Electrochemistry Russian Academy of Sciences (IPCE RAS), 31-4, Leninsky Prospect, 119071 Moscow, Russia; bazhanov.dmitriy@mail.ru (D.A.B.); ulianan@rambler.ru (U.V.N.); khasbiullin@techno-poisk.ru (R.R.K.); budylin_nikita@mail.ru (N.Y.B.); dr.evermakova@phyche.ac.ru (E.V.E.)

**Keywords:** additive manufacturing, crosslinking agent, photopolymerization, rheology, structure formation

## Abstract

The article presents the results of a study of the possibility of using heat-treated ethylene-vinyl acetate copolymer (EVA) as a thermoplastic modifier in a photosensitive composition based on tert-butyl acrylate (tBA). The use of such a modifier in 3D printing compositions is important for improving their physical and mechanical properties at low temperatures. An attempt was also made to use EVA as a polymer chain brancher. The molecular structure of the components and their compositions, rheology, curing kinetics, and phase organization of photocured systems were studied using FTIR and NMR spectroscopy, spectrophotometry, rheometry, Photo-DSC, and scanning electron microscopy. It was found that heat treatment of EVA allows the formation of single C=C bonds in macromolecules, which are necessary for a potential crosslinking agent with tBA. It was shown that EVA effectively functions as a thickener and modifier: with an increase in the modifier concentration, the nature of the composition flow changes from Newtonian to pseudoplastic, the rate of the photochemical polymerization reaction decreases, and the degree of conversion of the system decreases. However, the formation of a heterogeneous phase structure and the absence of a continuous spatial network of chemical bonds prevent the use of EVA simultaneously as a functional additive and crosslinking agent.

## 1. Introduction

There are many additive manufacturing (AM) processes that use different principles to construct physical objects. In 3D printing, in methods that use the principle of photopolymerization [1,2,3,4,5,6,7,8,9,10], as a result of the curing process, the initially “liquid” photosensitive composition, due to the initiation and occurrence of a photochemical polymerization reaction of components by a radical or cationic mechanism [1,2,3,4,6,8,9] under the influence of ultraviolet (UV) or visible (Vis) radiation from a 3D printer, is transformed into a “solid” polymer [2,3,5,6,9,10]. Moreover, the possibility of forming a three-dimensional network of chemical bonds, as well as its density, significantly influences the properties of the resulting material [8,9]. In order to form a unified polymer structure characterized by a dense network, necessary to impart good physical and mechanical properties to a 3D printed object, as well as high chemical [2,9] and thermal stability, crosslinking agents and/or active diluents [1,2,4,8,9] are included in the formulation of the photosensitive composition. The spatial network of chemical bonds in the photopolymer composition implements the so-called “shape memory effect” (SME), which opens up new possibilities for the use of 3D-printed products [4,5,8].

Acrylate and methacrylate monomers/oligomers are mainly used as the basis for photosensitive compositions for 3D printing [1,2,3,4,5,6,8,9]. Due to the limited choice of components for low-viscosity photosensitive compositions (LPSC), typical representatives are methyl methacrylate (MMA) [2], tert-butyl acrylate (tBA) [8], 1,6-hexanediol diacrylate (HDDA) [3,6,11], diethylene glycol diacrylate (DEGDA), 2-hydroxyethyl methacrylate (HEMA) [2] and triethylene glycol dimethacrylate (TEGDMA) [2,9]. The monomers MMA, tBA, and HEMA have only one acrylic group with an unsaturated bond in their chemical structure, therefore, they are not able to form a spatial network during photocuring and polymerize as a linear polymer with thermoplastic properties, soluble in polar solvents [4,9]. To branch the chain and form a network topology, it is necessary to use one or more monomers/oligomers with bi- or higher functionality, or to use crosslinking agents with C=C double bonds [1,2,4]. Thus, the use of tBA together with bifunctional acrylates allows for the production of “smart” 3D products that have “switching segments” and “network nodes” in their structure and, accordingly, exhibit an SME [8].

Acrylate-based LPSCs have significant drawbacks: high shrinkage during curing, which affects the geometric accuracy of 3D-printed objects [4,9]; anisotropy of properties and reduced interlayer adhesion in the resulting material, which leads to low physical and mechanical properties; high volatility and toxicity of components in the uncured state; and the ability to release unreacted substances from the cured material [2,3,4]. Therefore, basic LPSCs require modification by adding special additives (“modifiers”) of various natures in the uncured state [1,2,3,4].

Ethylene-vinyl acetate copolymer (EVA) can act as a potential modifying additive for acrylate-based LPSCs. This is primarily due to its thermoplastic properties and low glass transition temperature [12,13]. Therefore, regardless of the vinyl acetate (VA) content, unlike polymerized acrylates, EVA exhibits plastic deformation upon fracture and does not undergo brittle fracture even at low temperatures. This allows using EVA as a thermoplastic modifier to improve the crack resistance of composite materials and extend the operating temperature range of its products to lower temperatures. Secondly, there are a number of active patent documents [14,15,16,17,18,19] describing the compositions of UV-curable adhesives and coatings based on EVA and acrylate monomers/oligomers. This may indirectly indicate the good solubility of EVA in many acrylates, since, as noted in the cited documents, the developed adhesives and coatings stably cure under irradiation, forming a homogeneous structure. Thirdly, the ability of EVA to form unsaturated double bonds upon heat treatment or exposure to UV radiation was reported in [13,20]. It was noted that the longer the treatment, the greater the effect, and the highest results were demonstrated by EVA with a VA content of 40% (EVA40). However, prolonged exposure can lead to the destruction of the copolymer. When acrylate is irradiated in the presence of EVA, the presence of C=C double bonds can lead to their joint crosslinking into a single network, which allows EVA to be used as a crosslinking agent. A number of authors [13,21,22,23] also point out the reactivity of EVAs, which, under the influence of UV radiation and/or elevated temperatures, exhibit their own polymerization in the presence of peroxide or photoinitiators (PI). Fourth, it has long been known that the viscosity of polymer solutions increases with increasing polymer content. Accordingly, EVA, when dissolved in acrylates, can act as a thickener for photopolymer compositions.

Previously [24], the influence of several EVAs and low-density polyethylene (LDPE) on diffusion processes occurring in an uncured tBA-based photosensitive mixture was studied across the entire concentration range at different temperatures. Using the example of the diffusion of components in simple binary mixtures of “thermoplastic modifier—photosensitive monomer”, it was found that EVA40 is completely dissolved in tBA at room temperature, and the binary mixture was characterized by the highest interdiffusion coefficient. Therefore, the use of EVA40 as a polyfunctional additive with combined action for tBA-based LPSCs is of interest. The use of this copolymer as a thermoplastic modifier should improve the physicomechanical properties by lowering the glass transition temperature of the composite material. Furthermore, heat-treated EVA40 (m-EVA40) can act as a crosslinking agent due to the presence of C=C double bonds. At the same time, EVA40 is a thickener, which allows for regulating the viscosity of the photosensitive composition.

In [24], the fundamental basis necessary for the creation of photosensitive compositions modified with a thermoplastic for photopolymerization-based 3D printing methods was laid. The aim of this work is to evaluate the potential of m-EVA40 as both a thermoplastic modifier and a crosslinking additive in tBA-based photosensitive compositions for 3D printing.

## 2. Materials and Methods

### 2.1. Materials

The following substances were used as initial components. Monofunctional acrylate monomer—tBA, containing 15 per million (ppm) inhibitor 4-methoxyphenol from Acros Organics BVBA (Geel, Belgium). According to the manufacturer, the inhibitor is added to stabilize the monomer during storage. PI of radical polymerization—phenylbis (2,4,6-trimethylbenzoyl) phosphine oxide (BAPO). Commercial PI brand Irgacure 819 was obtained from Shanghai Macklin Biochemical Co., Ltd. (Shanghai, China). Thermoplastic polymer—EVA40. Commercial copolymer HANWHA EVA1540 was purchased from Hanwha Solutions Corporation (Seoul, Republic of Korea) and is a random product of radical copolymerization.

**Figure 1:**
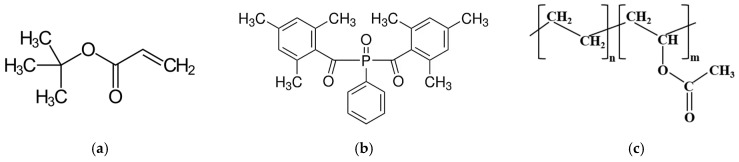
Structural chemical formulas of the used objects: (**a**) tBA; (**b**) BAPO; and (**c**) EVA40.

Figure 1 shows the structural chemical formulas of the material used. The initial commercial substances were characterized by various physicochemical methods. Information on their properties is summarized in Table 1.

### 2.2. Sample Preparation

#### 2.2.1. Preparation of the Initial Components

The EVA40 in its initial state was granules with a diameter of about 3–4 mm and was subjected to modification. The modification was carried out by heat treatment with the aim of forming C=C double bonds in place of VA units in the copolymer macromolecules, as described in [13,20]. Heat treatment was carried out by hot-pressing EVA40 granules on a laboratory hydraulic press at a temperature of 90 °C (40 °C higher than the melting point of EVA40 according to Table 1) in air for 2 min and with a force of 15 MPa. The processing conditions were determined by the mode of obtaining m-EVA40 under real conditions by the extrusion method. A polytetrafluoroethylene film was used as an anti-adhesive substrate. As a result of hot pressing, m-EVA40 film was obtained, which was slowly cooled to a room temperature of ~25 °C. The thickness of the pressed films was ~110 μm, the values of which were obtained using the coating thickness meter Easy-Check FE (List-Magnetik Dipl.-Ing. Heinrich List GmbH, Leinfelden-Echterdingen, Germany). The heat treatment mode for EVA40 was selected so that the resulting pressed m-EVA40 samples were not subject to a high degree of decomposition, but formed C=C bonds and, at the same time, retained the original structure of EVA, which is important for its complete solubility with tBA.

The pressed m-EVA40 films used later for the preparation of Modelling Photosensitive Compositions (MPSC) were pre-cut using a sterile scalpel along a template into approximately 3 × 3 mm plates. This was performed to increase the specific surface area and, accordingly, to increase the rate of dissolution of the modified copolymer in the acrylate monomer during mixing.

Commercial monomer tBA and PI BAPO were used as received from the manufacturer.

#### 2.2.2. Preparation of MPSCs

Five MPSCs were used in the study, one of which did not contain m-EVA40 and four with different m-EVA40 contents. All MPSCs were prepared in stoichiometric mass ratios, expressed as mass parts (mass. p.). This approach was used to proportionally reduce the tBA concentration relative to the BAPO concentration. The composition of the studied MPSCs and their corresponding weight percentages (wt.%) are given in Table 2.

The components were weighed in air on an OHAUS Adventurer Pro AV 114C laboratory scale (OHAUS Corporation, Parsippany, NJ, USA) with a maximum permissible error of ±0.0002 g in 4 mL amber glass bottles. Mixing was performed on a DLAB MS-H280-PRO laboratory magnetic stirrer with heating and a PT1000A temperature sensor (DLAB Scientific Co., Ltd., Beijing, China) for 30–90 min, depending on the amount of introduced m-EVA40. During mixing, the bottle was hermetically sealed with a screw cap without holes, which was made of polypropylene and had a red polytetrafluoroethylene septum for additional sealing. After mixing the components, all bottles with MPSCs were stored closed in a dark, thermostatically controlled cabinet at a temperature of 0 °C.

### 2.3. Methods

#### 2.3.1. Attenuated Total Reflectance—Fourier Transform Infrared (ATR-FTIR)

Samples of the initial EVA40, the obtained m-EVA40, the original tBA monomer, and all the prepared MPSCs before and after photocuring were examined using a Nicolet iN10 FTIR microscope (Thermo Scientific, Waltham, MA, USA) in the spectral region of 4000–675 cm^−1^ (the lower limit is determined by the ability of the germanium crystal of the ATR). Infrared (IR) spectra were obtained by ATR with the accumulation of 128 scans and with a resolution of 4 cm^−1^ at a room temperature of ~25 °C. Using Omnic 9 software (Thermo Scientific, Waltham, MA, USA), all IR spectra were transformed from transmittance to absorbance with automatic baseline correction.

#### 2.3.2. Nuclear Magnetic Resonance (NMR)

To confirm the FTIR spectroscopy results for the obtained m-EVA40, the liquid NMR spectroscopy method was used by a Bruker Avance III spectrometer (Bruker Corp., Billerica, MA, USA) with an operating frequency of 600 MHz. The ^13^C-NMR spectrum was recorded at a frequency of 150.9 MHz and a room temperature of ~25 °C using the residual solvent signal as an internal reference. The carrier liquid was deuterated chloroform (chloroform-*d*1), in which pre-cut m-EVA40 plates were dissolved. The mass fraction of the sample in the chloroform-*d1* solution was 1.0 g/mL. The internal reference of the spectrometer was tetramethylsilane (TMS, Me_4_Si), the chemical shift (*δ*) in ppm of which was taken as zero. The obtained ^13^C-NMR spectrum was given relative to TMS. All peaks in the spectrum were assigned according to the analysis of works [25,26,27,28,29], in which EVAs with different VA contents were previously studied by liquid and solid-state NMR methods.

#### 2.3.3. Spectrophotometry

Continuous absorption spectra of the tBA monomer and the obtained m-EVA40 were recorded using a highly sensitive fiber-optic UV–Vis spectrophotometer, Avantes AvaSpec-ULS2048CL-EVO (Avantes B.V., Apeldoorn, The Netherlands), equipped with a balanced deuterium halogen light source AvaLight-DH-S (Avantes B.V., Apeldoorn, The Netherlands). Scanning was performed in air in the absorption mode in the spectral range of 200–1100 nm. Quartz cuvettes with an optical path length of 1 cm were used for measurements at a room temperature of ~25 °C for 10 min. Continuous absorption spectra for each sample were recorded at least three times to ensure reproducibility of the results.

#### 2.3.4. Rheometry

Rheological study of uncured MPSCs was performed using a rotational viscometer (rheometer) HAAKE RheoStress 1 (Thermo Electron GmbH, Karlsruhe, Germany) connected to a HAAKE F6 circulation thermostat (Thermo Electron GmbH, Karlsruhe, Germany). Rheological properties of unmodified MPSCs were studied using a cone–plate sensor system (sensor titanium C35/2 Ti with diameter 35 mm and cone angle 2°, measuring plate steel MPC35 with diameter 36 mm and standard distance 0.105 mm). The modified MPSCs were tested using a plate–plate sensor system (sensor titanium PP20 with a diameter of 20 mm, measuring plate steel MP20-E with a diameter of 20 mm and standard distance of 1 mm). The tests were carried out in the controlled shear rate (CR) mode with stepwise reaching of points with a stationary value (“CR Rotation Step mode”) in the shear rate range of $\dot{\gamma }$ = 0.1–100 s^−1^ at a temperature of 20 °C. The maximum waiting time to achieve stationary conditions was 60 s. The choice of the test mode and parameters was determined by the recommendations in the literature [30]. The experiment was started after preliminary thermostatting and equilibration of the sample in the measuring system for 2 min at the test temperature. Taking into account the influence of shear history [30], the rheological properties of all MPSCs were determined on three independent samples to obtain more reliable results.

#### 2.3.5. Differential Scanning Photocalorimetry (Photo-DSC)

Thermal effects of the in situ photochemical polymerization reaction of the studied MPSCs were recorded using a NETZSCH DSC 204 F1 Phoenix differential scanning photocalorimeter (Netzsch-Geratebau GmbH, Selb, Germany) with a Thorlabs OSL2 Fiber Illuminator prefix-light source (Thorlabs Inc., Newton, NJ, USA). The Photo-DSC device was developed on the basis of a conventional DSC device by analogy with previously published works [6,31]. A detailed description of the modification of the conventional DSC device design in order to transform it into a Photo-DSC is presented in the Supplementary Materials (Figures S1–S5).

Since temperature and environment affect the thermal effect and rate of photochemical polymerization reaction [6,7,11,31,32], all measurements by this method were carried out in isothermal mode in an inert atmosphere. A 35 ± 2 mg sample of MPSC was weighed in air in an open aluminum crucible with a volume of 40 μL. The crucible was covered with silicate glass to prevent evaporation of tBA. Then the crucible with the sample was placed in the crucible holder of the furnace of the device. The reference crucible was empty. The crucible with the sample was thermostatted for 4 min at a temperature of 30 °C in an argon atmosphere at a flow rate of 60 mL/min. After 4 min, the attachment lamp was turned on. Irradiation was carried out continuously for 10 min. The power factor (PF) of the lamp radiation was set by the light adjustment knob to 50%, which corresponds to the intensity of incident lamp radiation (*I*_0_) on the sample surface of ~0.0845 mW/cm^2^, according to the readings of the UV radiometer “TKA-PKM” (NTP TKA LLC, St. Petersburg, Russia) in the UVA range. After cessation of irradiation, the crucible with the sample was kept in the furnace of the device for another 10 min in order to ensure completion of photopolymerization. Sample losses after the experiment did not exceed 10 wt.%. To obtain reliable results, three independent experiments were carried out for each MPSC.

#### 2.3.6. Construction of Isotherms of Photochemical Polymerization Reaction and Integral Kinetic Curves of MPSC Photocuring Process

The construction of photochemical polymerization reaction isotherms (Photo-DSC isotherms) for the studied MPSCs is carried out using the obtained Photo-DSC traces in *dq*/*dt*—*t* coordinates.

**Figure 2:**
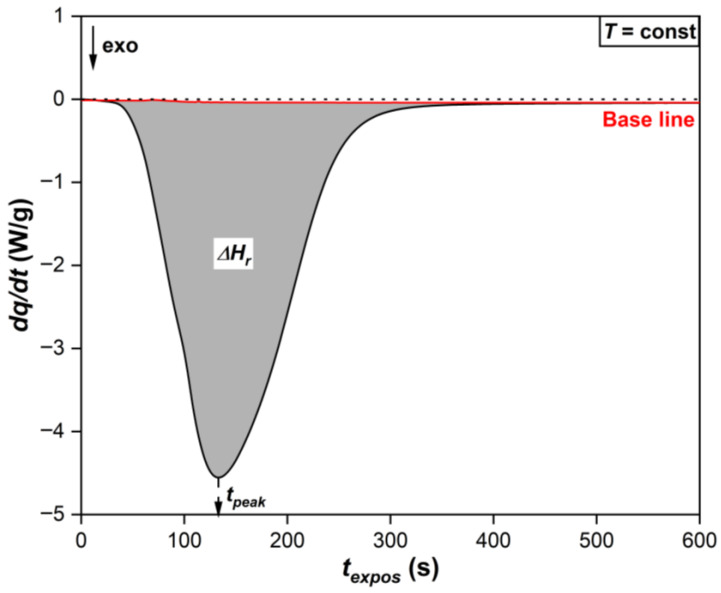
Example of baseline subtraction and calculation of the area under the exothermic peak curve on the Photo-DSC isotherm.

In Photo-DSC experiments [6,31], a thermal imbalance usually occurs, which results in the heat flow (*dq*/*dt*) being higher than the initial value after cessation of irradiation and arrest of the radical chemical reaction. The causes of thermal imbalance include both uneven absorption of UV and residual near-infrared radiation from the lamp by the sample crucible and the reference crucible, as well as imperfect design of the device, which causes thermal noise and instability of the baseline. Therefore, to obtain the true *dq*/*dt* coming only from the chemical reaction zone, it is necessary to subtract the baseline from the initially recorded heat flow. That is, to adjust the baseline, as shown in Figure 2. One option for obtaining the baseline *dq*/*dt* is to re-irradiate a fully cured sample, which does not exhibit heat release as a result of photopolymerization [6,31].

The experimental value of the heat of reaction of photochemical polymerization is calculated by finding the corresponding enthalpy of reaction, equal to the area under the Photo-DSC curve (gray shaded area) relative to the baseline (red curve), as shown in Figure 2 [6,7,33]:

(1) $$Q_{exp}=-∆H_{r},\left[J/g\right],$$

where *Q_exp_* is the experimental value of the amount of heat released during photopolymerization of the photosensitive composition, [J/g]; Δ*H_r_* is the enthalpy of the chemical reaction of photopolymerization of the photosensitive composition, [J/g].

The time to reach the exothermic peak (*τ_peak_*) is taken as the maximum on the Photo-DSC isotherm.

The recorded data on *dq*/*dt* during the chemical reaction allows for the construction of integral kinetic curves of the photocuring process of MPSCs. They represent the dependence of the degree of conversion (*α*) on time (*t*) under the assumption that *α = α*(*t*) [11,32]. The Photo-DSC method assumes that during the photocuring experiment, the measured heat flow is proportional to the conversion rate of MPSC or the polymerization rate of its reactive components at a constant temperature (*T*) [11,32,33] according to the following equation:

(2) $$\frac{d\alpha }{dt}=\frac{1}{Q_{theor}}{\left(\frac{dq}{dt}\right)}_{T},[1/s],$$

where *dα*/*dt* is the conversion rate, [1/s]; *Q_theor_* is the maximum possible (theoretical) amount of heat released during the complete chemical reaction, [J/g]; (*dq*/*dt*)*_T_* is the isothermal heat flow at time *t*, [W/g].

Then *α* for MPSC at any time *t* is determined by relating the amount of heat released from the reaction zone at a given time to the maximum possible (theoretical) amount of heat of the photochemical polymerization reaction [6,7,11,33]:

(3) $$\alpha \left(t\right)=\frac{1}{Q_{theor}}\int_{0}^{t}{\left(\frac{dq}{dt}\right)}_{T}dt=\frac{Q_{t}}{Q_{theor}},\left[a.u.\right],$$

where *Q_t_* is the amount of heat released from the reaction zone at time *t*, [J/g].

The final degree of MPSC conversion obtained as a result of photopolymerization can also be estimated using the following equation [31,32]:

(4) $$\alpha =\frac{Q_{exp}}{Q_{theor}}\cdot 100,\left[\%\right].$$

According to various data [32,33], it is known that during complete polymerization (*α* = 1) of monofunctional acrylate, about 80–86 kJ/mol of heat is released. Consequently, the maximum possible amount of heat (theoretical) released during 100% polymerization of 1 g of *N*-functional acrylate is calculated as follows [32]:

(5) $$Q_{theor}=\frac{Q_{A}\cdot N}{M_{A}}=\frac{83000\cdot N}{M_{A}},\left[J/g\right],$$

where *Q_A_* is the molar heat released during complete polymerization of monofunctional acrylate, [J/mol]; *N* is the functionality of acrylate (the number of acrylic groups in the molecule); *M_A_* is the molecular weight of acrylate, [g/mol].

In the case of MPSCs containing the thermoplastic modifier m-EVA40, Equation (5) needs to be adjusted as follows:

(6) $$Q_{theor}=\left(\frac{83000\cdot N_{tBA}}{M_{tBA}}\right)\cdot \omega_{tBA}+\left(\frac{Q_{m-EVA40}\cdot N_{m-EVA40}}{M_{m-EVA40}}\right)\cdot \omega_{M-EVA40},\left[J/g\right],$$

where *N_tBA_* is the number of acrylic groups in the tBA molecule; *M_tBA_* is the molecular weight of tBA, [g/mol]; *ω_tBA_* is the mass fraction of tBA in the photosensitive composition; *Q_m-EVA40_* is the molar heat released during complete polymerization of C=C double bonds in the m-EVA40 macromolecule, [J/mol]; *N_m-EVA40_* is the number of C=C double bonds in the m-EVA40 macromolecule; *M_m-EVA40_* is the molecular weight of m-EVA40, [g/mol]; *ω_m-EVA40_* is the mass fraction of m-EVA40 in the photosensitive composition.

However, since the concentration of thermoplastic modifier m-EVA40 in all MPSCs is relatively low, we neglected, as a first approximation, the contribution of heat from m-EVA40 to the maximum theoretical heat of photochemical polymerization reaction corresponding to MPSC. Therefore, for calculations we used Equation (5) and considered that the kinetics of photocuring (which is usually equal to the kinetics of photopolymerization [32]) of all MSCs is equal to the kinetics of consumption of only the double bond H_2_C=CH– in tBA.

#### 2.3.7. Scanning Electron Microscopy (SEM)

The phase structure of the cured MPSCs was studied using scanning electron microscopy on a JSM 6060A (JEOL, Ltd., Tokyo, Akishima, Japan). Sample preparation consisted of obtaining low-temperature chips of the studied objects and magnetron sputtering of a conductive gold layer on their surface in a DSR (Nano-Structured Coatings Co., Tehran, Iran) device. Samples were photographed at an accelerating voltage of 15 kV, and images were obtained using secondary electron emission.

## 3. Results and Discussion

**Figure 3:**
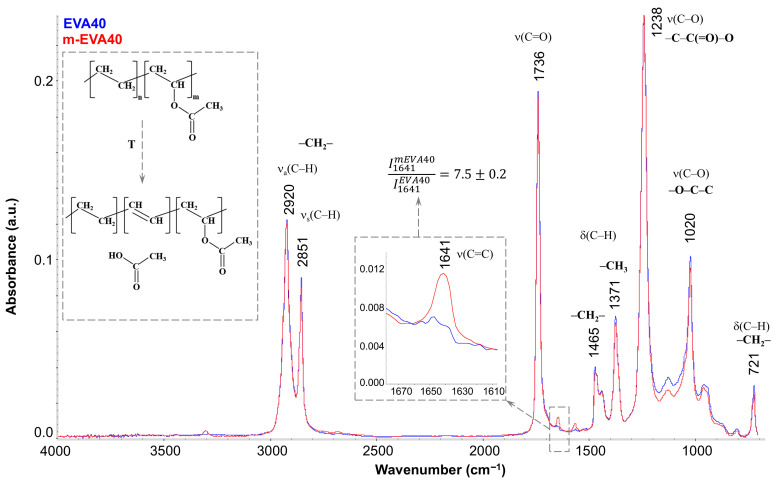
FTIR spectrum at 25 °C for EVA40 (blue spectrum) and m-EVA40 (red spectrum).

Figure 3 shows the FTIR spectra of EVA40 (blue spectrum) and m-EVA40 (red spectrum). It is seen that they have a standard appearance for the IR spectra of EVAs [34,35] and are characterized by the presence of the main absorption bands associated with the C–H stretching vibrations in the region of 3000–2800 cm^−1^, stretching vibrations of the carbonyl group C=O at 1736 cm^−1^, and the stretching vibrations of C–O in the –C–C(=O)–O and –O–C–C groups (1238 and 1020 cm^−1^, respectively), as well as with the deformation vibrations of these groups. The only difference between the spectra of EVA40 and m-EVA40 is the presence of a noticeable low-intensity peak at 1641 cm^−1^ in the latter (separately enlarged in the inset of Figure 3). Quantitative analysis showed that the intensity of this peak increases more than sevenfold for m-EVA40. The presence of absorption peaks in this region has been recorded for EVAs before [13,36], and their intensity increases noticeably with increasing VA content in EVA. Opinions are divided regarding the nature of this peak. Some authors [36] stated that there is a redistribution of the electron cloud in the VA group and the formation of stable hydrogen bonds between the oxygen of the carbonyl group and the hydrogen of the –CH_2_– group in the main hydrocarbon chain. Indeed, absorption peaks associated with deformation vibrations of hydroxyl groups and hydrogen bonds may appear in the 1600–1500 cm^−1^ region, but this requires the presence of noticeable absorption peaks in the region of stretching vibrations of –OH groups in the 3600–3100 cm^−1^ region. Other authors, under a number of conditions (exposure to elevated temperature, UV radiation, etc.) [13,37], recorded the splitting off of the entire VA group and the formation of a double bond between the carbons of the main chain. In this case, the absorption band at 1641 cm^−1^ coincides well with the absorption region of C=C bonds. The confirmation of the formation of a C=C double bond with the elimination of CH_3_COOH (the conventional scheme is shown in the inset in Figure 3) is supported by the fact that during the heat treatment of EVA a slight smell of acetic acid is felt, and a slight decrease in the intensity of the 1736 cm^−1^ band (i.e., a decrease in the content of carbonyl groups) is observed in the IR spectrum, as well as a decrease in the intensity of the 1020 cm^−1^ peak and a general decrease in the intensity in the range of 1150–950 cm^−1^. It should be noted that this process is not spontaneous in nature, with a complete replacement of the VA monomer units, but only complicates the structure of the copolymer, in which, in addition to ethylene or VA fragments, C=C double bonds are added.

**Figure 4:**
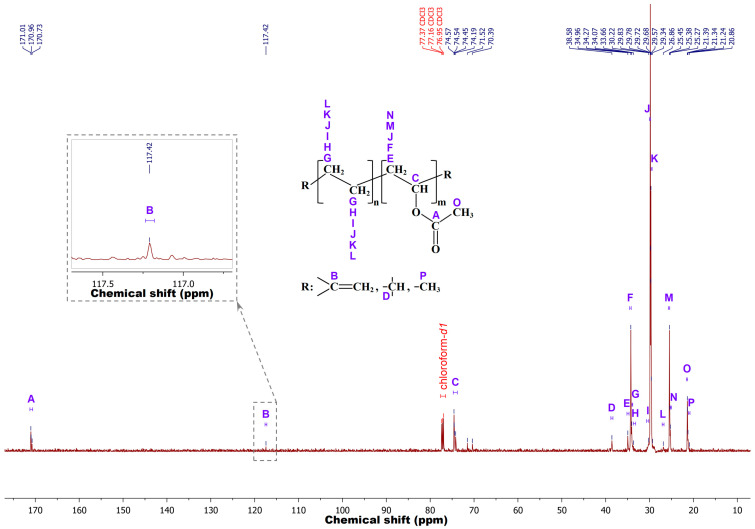
^13^C-NMR spectrum for m-EVA40 in chloroform-*d1* at 25 °С and 150.9 MHz.

To confirm the results obtained by FTIR spectroscopy, the chemical structure of m-EVA40 was additionally investigated by liquid NMR spectroscopy. Preliminary analysis was performed by proton magnetic resonance to record the ^1^H-NMR spectrum. However, the data obtained are not indicative, since, due to the high number of –CH_2_– groups in the chemical structure of EVA, the dynamic range of the NMR spectrometer is reached very quickly and the low-intensity signal from hydrogen at the double bond either does not have time to be recorded or is blurred against the background of other signals, turning into noise, or merges into a single peak with the CH–O– fragment of the VA unit. In this regard, ^13^C-NMR is advisable for better resolution. Figure 4 shows a typical ^13^C-NMR spectrum recorded for the m-EVA40 sample in chloroform-*d1* at room temperature. The spectral line sequences according to [25,26,27,28,29] are as follows (solvent signals are omitted)—^13^C-NMR (150.9 MHz, Chloroform-*d*1, *δ* in ppm): 171.01–170.73 (A), 117.42 (B), 74.57–74.19 (C), 71.52 (false positive), 70.39 (false positive), 38.58 (D), 34.96 (E), 34.27 (F), 34.07 (G), 33.66 (H), 30.22 (I), 29.83–29.78 (J), 29.72–29.57 (K), 29.34 (false positive), 26.86 (L), 25.45–25.38 (M), 25.27 (N), 21.39–21.34 (O), and 21.24–20.86 (P). It is evident that in m-EVA40, in addition to the main ethylene and VA groups, there are branches at the methine point (signal D) and methyl groups –CH_3_ (signal P). The methyl group is probably the terminal functional group in this copolymer, as shown in the work [25]. There are resonance signals related to carbon at the C=C bond (signal B), indicating the presence of C=C bonds in m-EVA40. The experimental data obtained by the liquid NMR spectroscopy method correlate with FTIR spectroscopy results.

Thus, both spectroscopic methods confirm the formation of unsaturated C=C double bonds at the site of the VA monomer unit along the EVA40 chain after a short thermal treatment due to the elimination of acetic acid [13,20]. Based on the obtained results, as well as after analyzing a number of works [13,21,22,23], it can be concluded that m-EVA40 is potentially capable of crosslinking with the tBA monomer to form a three-dimensional spatial network of chemical bonds. This can occur due to the opening of the C=C bond after the attack of active particles of decomposed PI (BAPO in our case) to form the corresponding active center [7,13]. Consequently, m-EVA40 in this case should have a certain effect on the kinetics of photopolymerization and rheokinetics of photocuring of modified MPSCs.

**Figure 5:**
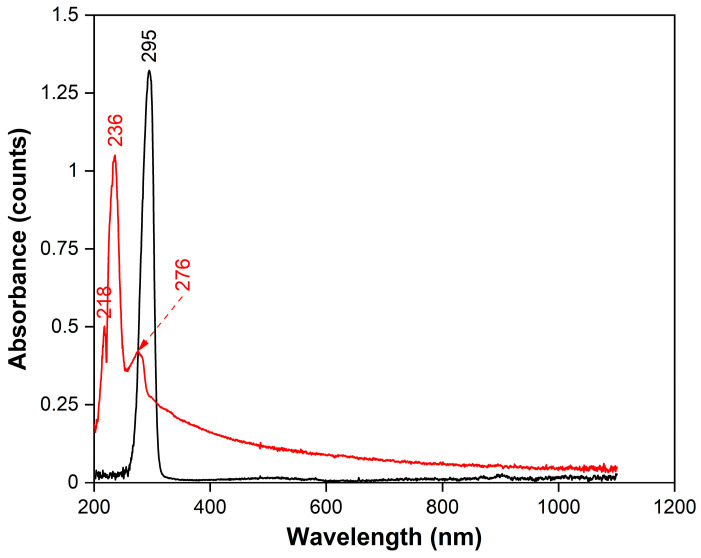
Continuous absorption spectrum at 25 °C for tBA (black spectrum) and for m-EVA40 (red spectrum).

To obtain information on the absorption of radiation in the UV−Vis range, continuous absorption spectra were recorded for tBA and m-EVA40 by spectrophotometry (Figure 5). It is evident that tBA (black spectrum) has only one absorption peak in the range of 200–400 nm, with a maximum at 295 nm. It is associated with the presence of the H_2_C=CH– group in the monomer molecule and its interaction with the spectrophotometer radiation, namely with the process of absorption of photons of the corresponding frequency. In the case of m-EVA40 (red spectrum), due to its complex chemical structure, a fairly wide absorption zone is observed in the spectrum, the largest part of which is within 200–500 nm. At the same time, three peaks are recorded in the UVB range, with maxima at 218, 236, and 276 nm, respectively. These observations also confirm the appearance of a C=C double bond in m-EVA40, since the electronic absorption spectra of substances with C=C bonds usually have absorption maxima in the range of 180−240 nm. It should be noted that the absorption range of tBA does not overlap with the effective absorption region of BAPO, which, according to [38], is in the region of 325–450 nm. Similarly, m-EVA40, according to [2] and spectrophotometry data, does not exhibit strong absorption in this region and does not affect the photoinitiation process of BAPO. Therefore, it is rational to use BAPO as a PI for modified MPSCs.

**Figure 6:**
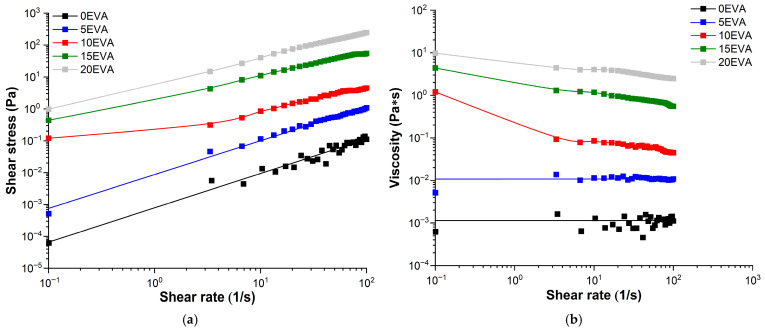
Rheological properties of MPSCs at 20 °C: (**a**) flow curves; (**b**) viscosity curves.

Based on the results of the rheological study, the flow and viscosity curves (rheograms) were obtained for all the prepared MPSCs (Figure 6a,b, respectively). The introduction of m-EVA40 into MPSCs leads to an increase in the *η* value in the range of $\dot{\gamma }$ = 0.1–100 s^−1^. Moreover, the addition of m-EVA40 in an amount of 10 mass. p. or more leads to a sharp increase in the dynamic viscosity and a change in the nature of the system flow from Newtonian to pseudoplastic in the studied range of shear rates [30]. In the absence of intermolecular interactions between the components in the modified MPSCs in the uncured state in polymer solutions at a certain concentration, the macromolecular chains form loops and chaotically entangle with each other, forming a fluctuation network of entanglements. At low polymer concentrations, individual macromolecules practically do not interact with each other, i.e., at a concentration of m-EVA40 < 10 mass. p. MPSC has a relatively low viscosity and shows Newtonian flow. With an increase in the modifier concentration, the entanglement and interweaving of macromolecular chains dissolved in tBA increase. And at a certain concentration of m-EVA40, a state with an irregular internal order arises, which is characterized by high viscosity at rest or under low shear stress. When the shear rate is increased to a value where the disorienting influence of chaotic Brownian motion is overcome, the macromolecular chains of m-EVA40 in solution unravel, stretch, and orient in the direction of the driving force. As a result of the orientation, an asymptotic decrease in viscosity is observed to a certain constant value—the “lowest Newtonian” viscosity [30].

Based on the rheometry results, the rheological behavior of MPSC with a content of *С_m-EVA40_* < 10 mass. p. in the composition obeys Newton’s law of viscous friction (Equation (7)), and with *С_m-EVA40_* ≥ 10 mass. p., as it is described by the Ostwald–de Waele power law (Equation (8)) [30]:

(7) $$\tau =\eta \cdot \dot{\gamma },\left[Pa\right],$$

(8) $$\tau =k\cdot {\dot{\gamma }}^{n},\left[Pa\right],$$

where *k* is the consistency index related to viscosity, [Pa∙s^n^]; *n* is the anomaly index (power index of nonlinearity).

**Figure 7:**
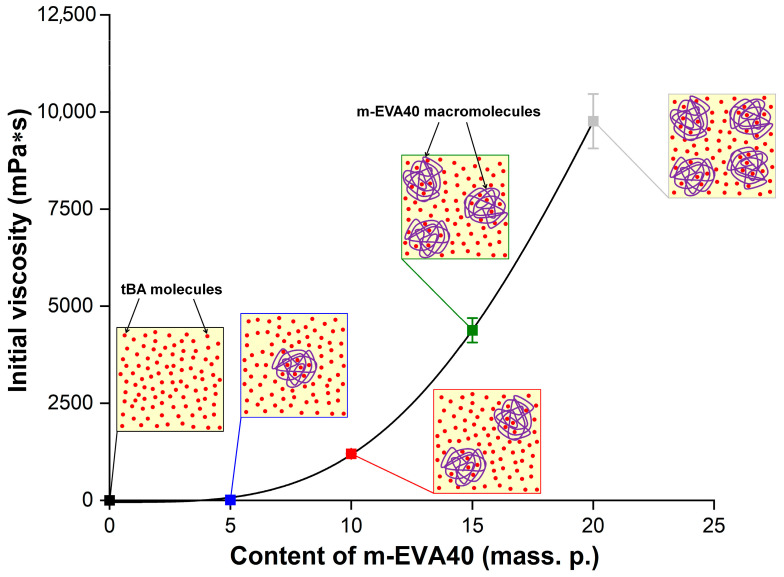
Dependence of the initial dynamic viscosity at 25 °C on the concentration of m-EVA40 in MPSCs. The insets in the figure schematically show the supramolecular structure of uncured MPSC of different content: the small red dots correspond to tBA molecules, and the purple coils correspond to m-EVA40 macromolecules.

The concentration dependence of the dynamic viscosity for MPSC at $\dot{\gamma }$ = 0.1 s^−1^ (*η*_0.1_) is shown in Figure 7, with a schematic illustration of the supramolecular structure described above. The obtained dependence of *η*_0.1_ as a function of *С_m-EVA40_*. Table 3 contains the experimental values of *η*_0.1_ and the initial shear stress at $\dot{\gamma }$ = 0.1 s^−1^ (*τ*_0.1_) for all MPSCs, as well as the parameters from Equations (7) and (8). It is evident that, compared to the unmodified MPSC, the modified system contains 20 mass. p. m-EVA40 and has a *η*_0.1_ value that is almost four decimal orders of magnitude higher. It should be noted that the dependence on *η*_0.1_(*C_EVA-40_*) is nonlinear. Introduction to MPSC 10 mass. p. and more thermoplastic modifier leads to a sharp rise in the curve.

Based on the conducted rheological study, it can be concluded that the introduction of m-EVA40 into the initial tBA-based LPSC allows an increase in its viscosity to the required level. Several publications [3,8,10] report that the viscosity of photosensitive compositions used in classical photopolymerization-based 3D printing methods should be at a level of 0.1 to 10 Pa·s. Thus, it can be argued that m-EVA40 acts as a thickener for MPSC and the introduction of this copolymer in an amount of 10–20 mass. p. allows an increase in the processability of the original system, as well as using it in photopolymerization-based 3D printing.

**Figure 8:**
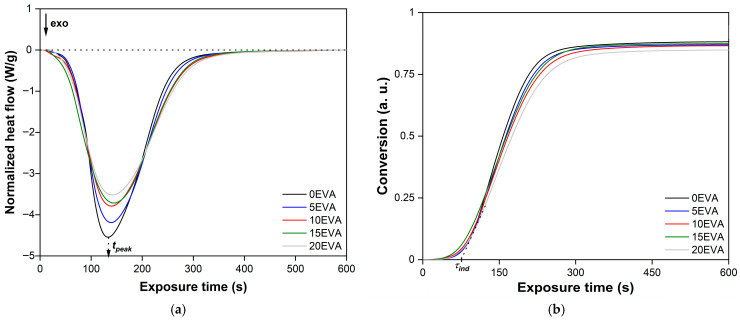
(**a**) Photo-DSC isotherms of MPSCs at 30 °C; (**b**) integral kinetic curves of MPSCs at 30 °C.

Figure 8 shows typical Photo-DSC isotherms of the photopolymerization process of the initial and modified MPSCs. It is evident that even with an m-EVA40 content of 20 mass. p. the system exhibits a stable photochemical polymerization reaction. The initial stage of photopolymerization of all MPSCs is characterized by an induction period (*τ_ind_*). After ~85 s, a sharp increase in the rate of the chemical reaction is observed on all isotherms, which was called the Trommsdorff–Norrish effect or the “gel effect” [31,32]. During the chemical reaction of photopolymerization, an exponential decrease in the heat flow from the reaction zone occurs, forming an extremum on the obtained curves, which is estimated as the time to reach the exothermic peak (*τ_peak_*). As a result, all curves reach a plateau within ~570 s after the start of irradiation, which is a sign of completion of the photochemical polymerization reaction corresponding to MPSC [31,32]. These observations are consistent with the results of studying the curing process of various acrylates and systems based on them [6,7,11,32,33]. The slowdown in the photopolymerization rate after reaching the peak value is explained by the fact that in MPSCs, viscosity increases over time during curing, the molecular weight increases, and gelation occurs. All this leads to a decrease in the mobility and a decrease in the diffusion of all components of the system, including reactive ones. Consequently, the rate of the photochemical polymerization reaction also decreases. In addition, the gradual consumption of C=C bonds, a decrease in the efficiency of BAPO as a result of photodegradation, and vitrification of the system at a later stage of the chemical reaction also contribute to a decrease in the photopolymerization rate until it stops. Another factor is associated with an increase in the glass transition temperature (*T_g_*) of the polymer formed during the reaction, which leads to vitrification of the corresponding MPSC as soon as *T_g_* exceeds the experimental temperature [5,6,7,21,22,23,24,25,26,27,28,29,30,31,32,33]. In the case of modified MPSCs, the rate of photochemical polymerization reaction is additionally affected by phenomena that may be related to the absorption and scattering of the lamp radiation from the attachment on the macromolecules of the thermoplastic modifier [5,11]. Based on the analysis of isotherms in *α*—*t_expos_* coordinates, integral kinetic curves of the photopolymerization process of the studied MPSCs were constructed (Figure 8b). It is evident that all curves have a classical S-shaped character [32]. The *α*(*t_expos_*) dependences clearly show *τ_ind_* and the inflection point corresponding to the maximum on the corresponding isotherm. These observations are an indication of the occurrence of an autocatalytic chemical reaction, which correlates with the results of studying the photopolymerization of other acrylate-based systems [6,7,11,32,33].

**Figure 9:**
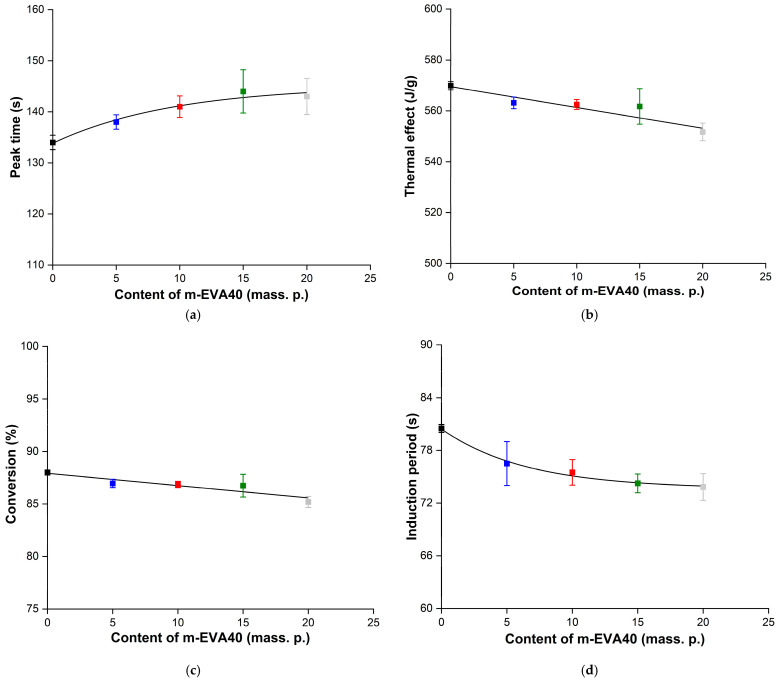
Effect of m-EVA40 concentration in MPSC on the following: (**a**) time to reach the exothermic peak upon irradiation; (**b**) thermal effect of photochemical polymerization reaction (per monomer); (**c**) degree of conversion (per monomer); and (**d**) induction period.

Figure 9a,b shows the dependence of the *τ_peak_* and *Q_exp_* values as functions of *C_m-EVA40_*, respectively. It is evident that the addition of m-EVA40 to the initial system results in a slight increase in *τ_peak_*, while the dependence of *τ_peak_*(*C_m-EVA40_*) is nonlinear and is described by a “saturation curve”. With an increase in the content of the thermoplastic modifier, a linear decrease in the experimental value of *Q_exp_* is also observed. Apparently, both facts indicate a slowdown in the autoacceleration of the ongoing photochemical polymerization reaction with an increase in the concentration of m-EVA40 in MPSCs. This can be explained by the fact that m-EVA40 macromolecules dissolved in the tBA monomer have a branched structure and are characterized by fairly large sizes [26] relative to the radicals and active centers formed from BAPO and then tBA at the stages of initiation and initial chain growth. In addition, as has already been said earlier, at a certain concentration of m-EVA40, a fluctuation network of entanglements arises in the system, leading to a sharp increase in the initial viscosity [30]. All this can create steric hindrances for the movement and diffusion of reactive particles in the volume of MPSC, reducing their mobility at the chain growth stage. At the same time, the thermoplastic modifier can contribute to the weakening of the intensity of incident radiation as a result of partial light absorption and/or light scattering on dissolved macromolecules [4,5,11,39]. In addition, in curing systems modified with thermoplastics, during the chemical reaction, it is possible to form heterogeneous structures at the interphase boundaries, where light scattering also occurs [40]. Therefore, despite the potential reactivity of m-EVA40 due to the presence of C=C double bonds in its chemical structure, in general, there is a decrease in the rate of the photochemical reaction of polymerization of modified MPSCs. The amount of heat released from the reaction zone also decreases, and the degree of conversion of the system drops. Figure 9c,d shows, respectively, the dependences of the *α* and *τ_ind_* values as the corresponding functions of *C_m-EVA40_*. It is evident that with the increase in m-EVA40 concentration from 0 to 20 mass. p. in MPSCs, there is a tendency to some decrease in both the degree of conversion and the induction period. The explanation for this was given above. Table 4 summarizes the results of the analysis of Photo-DSC isotherms and integral kinetic curves of the photopolymerization process of the studied MPSCs, and also provides the calculated values of the key parameters. All presented data were statistically processed, and the coefficient of variation does not exceed 3%.

**Figure 10:**
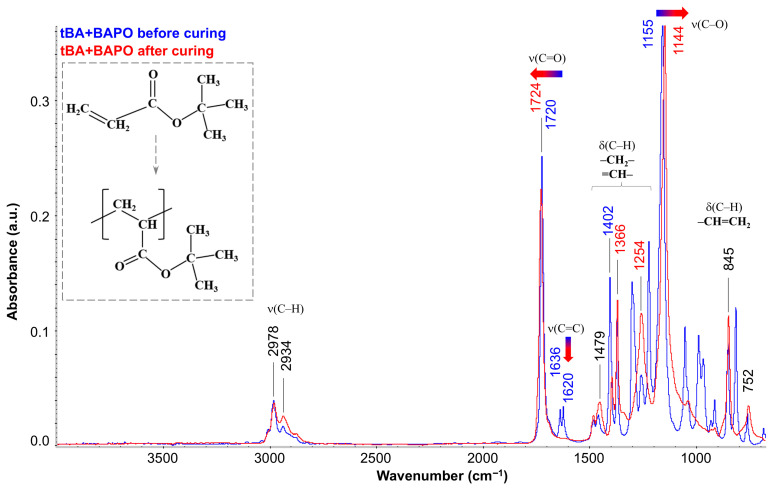
FTIR results at 25 °C: IR spectrum of tBA + BAPO before curing (blue spectrum) and after curing (red spectrum).

Figure 10 shows the FTIR spectra for tBA with BAPO before and after photopolymerization (blue and red spectra, respectively). It should be noted that in the preliminary experiment, the spectra of tBA were obtained and analyzed both without PI and in its presence. This experiment showed that adding 1 mass. p. BAPO to the monomer does not lead to noticeable changes in the spectrum, and, therefore, the presented spectrum for tBA + BAPO completely corresponds to the spectrum of pure tBA and shows absorption bands characteristic of it. The most intense of them are the peaks at 1720 cm^−1^ (stretching vibrations of the carbonyl group C=O) and 1155 cm^−1^ (stretching vibrations of the C–O bond). In addition, absorption bands of both the stretching vibrations of C–H bonds (range 3050–2800 cm^−1^) and the deformation vibrations of various groups (1450–1200 cm^−1^ and 1100–700 cm^−1^) can be observed. It is important to emphasize that at 1636 and 1620 cm^−1^ there is a characteristic double peak associated with the presence of a C=C double bond at the end of the initial monomer. After curing (transition from blue to red spectrum), the peaks of double bonds in poly-tBA completely degenerate (shown by the blue–red arrow), indicating complete conversion of the tBA monomer. Quantitative calculation according to the absorption band at 1620 cm^−1^ showed that the conversion degree reaches 97%. In addition, a shift in the peak of the C–O stretching vibrations at 1155 cm^−1^ to a new position at 1144 cm^−1^ (also shown by the arrow) can be observed, and the carbonyl peak also changes slightly and shifts to the left (from 1720 to 1724 cm^−1^). These changes in the spectrum are due to the fact that the opening of the acrylate double bonds leads to a change in the spatial arrangement of all neighboring groups. In this case, the intensity of the band at 2934 cm^−1^ (stretching vibrations of the –CH_2_– group) and 1479 cm^−1^ (deformation vibrations of the –CH_2_– group) increases, indicating the appearance of a carbon skeleton. At the same time, analysis of the band at 1366 cm^−1^, associated with deformation vibrations of the C–CH_3_ group, showed that no significant changes in the –CH_3_ groups were observed. Also, the absorption bands in the region of 1100–900 cm^−1^, which were associated with deformation vibrations of C–H at the C=C double bond, disappear. Thus, it can be stated that during the curing process, the acrylate double bonds in tBA are completely opened to form the main macromolecular chain, where the tert-butyl part, which has changed its spatial position (but not structure), becomes the side group of the resulting linear polymer poly-tBA (the schematic diagram is shown in the inset in Figure 10).

**Figure 11:**
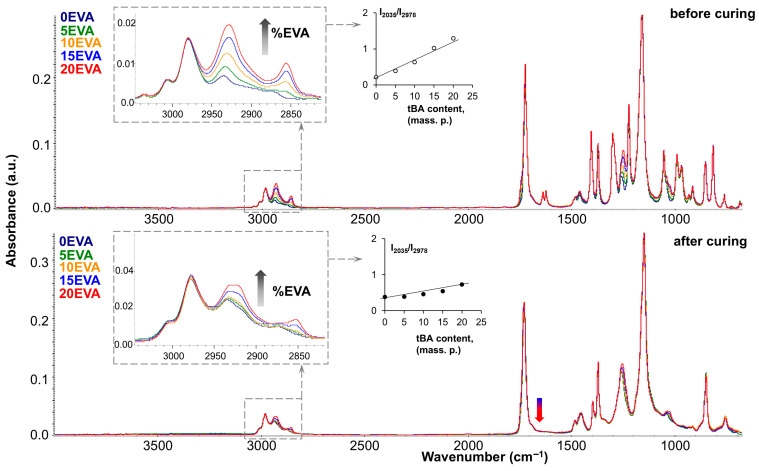
FTIR results at 25 °C: IR spectra of MPSCs before curing (**top**) and after curing (**bottom**).

Figure 11 shows the FTIR spectra of the original and modified MPSCs before and after photocuring (top and bottom, respectively). As can be seen, the mixed spectra almost completely correspond to the spectrum of pure tBA monomer (dark blue spectrum). However, the introduction of m-EVA40 can be tracked by the increase in the intensity of such characteristic absorption bands as the stretching vibrations of the –CH_2_– group, which appear in the region of 2950–2800 cm^−1^. The inset on the left shows that the higher the content of the thermoplastic modifier in the system, the higher the intensity of these absorption peaks in the spectrum. A quantitative analysis of the 2930 cm^−1^ band growth was performed, and a plot of its change with increasing m-EVA40 content was plotted (shown in the center of Figure 11). The linear growth of this absorption band, both before and after curing, indicates that increasing the m-EVA40 content in the mixture does not affect the curing process of tBA. Such an effect can be observed for the absorption band in the region of 1250 cm^−1^ (stretching vibrations of C–O in the –C–C(=O)–O group), the intensity of which increases with the increase in the content of m-EVA40 in MPSC, and this peak also tends to shift to the right due to the fact that for pure m-EVA40 its position corresponds to 1238 cm^−1^. The carbonyl peak (1730–1720 cm^−1^) also increases slightly in intensity and broadens to the left. This is due to the fact that similar absorption bands of the two components (tBA and m-EVA40) in the original spectra have a slightly different position in wavenumber due to the influence of neighboring groups. For example, the carbonyl peak of m-EVA40 is localized at 1736 cm^−1^, while for tBA it is at 1720 cm^−1^. Accordingly, both peaks appear during mixing, and the corresponding changes in the spectrum can be seen in total. By additionally processing the IR spectra of the initial components tBA and m-EVA40, through their mathematical addition, simulated mixed spectra were obtained, which were completely identical to the experimentally obtained mixed spectra for MPSCs. All this proves that no interactions or transformations occur during the mixing of tBA and m-EVA40. After photocuring, the mixed spectra also differ only in the intensity of the characteristic bands of m-EVA40, as was the case for the mixed spectra before curing. This suggests that m-EVA40 is potentially present before and after curing as an additional component that does not participate in crosslinking. It is important to emphasize that the obtained mixtures were obtained using m-EVA40, which is characterized by the presence of C=C double bonds, and the absorption bands of C=C bonds in the mixed spectra after curing completely disappear (indicated by the blue–red arrow in Figure 11). This may be the result of the fact that there are too few C=C double bonds in m-EVA40, and its potential participation in copolymerization with tBA does not lead to the formation of a dense network.

The swelling method showed that the cured samples of all MPSCs were completely dissolved in tetrahydrofuran and xylene within 24 h at a room temperature of ~25 °C. This confirmed our assumption based on the FTIR spectroscopy results. Therefore, it is fair to emphasize that the content of formed C=C double bonds after thermal treatment of EVA40 in the specified pressing modes at a concentration of thermoplastic modifier in MPSC up to 20 mass. p. is insufficient to perform crosslinking and form a dense three-dimensional network of chemical bonds.

**Figure 12:**
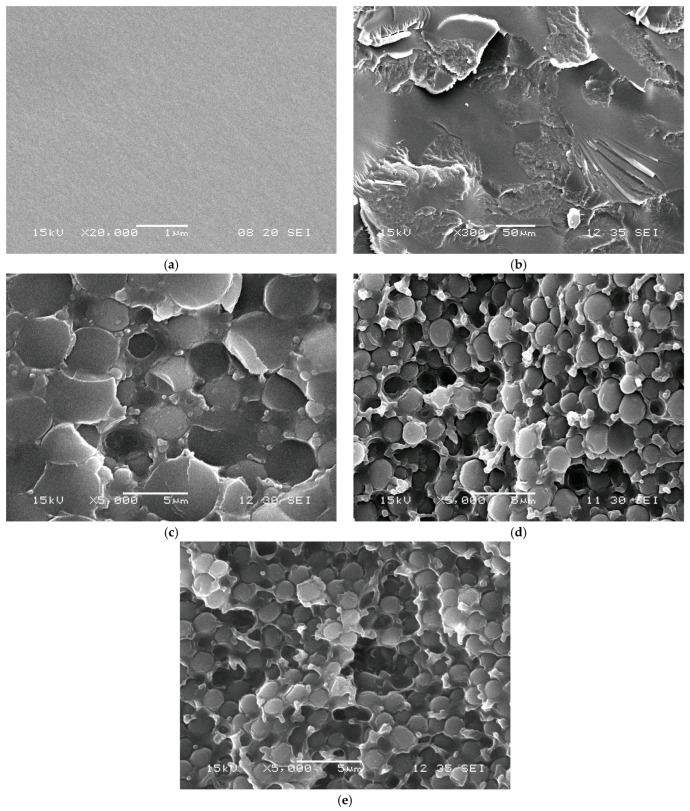
The SEM images of cured MPSCs with different m-EVA40 contents: (**a**) 0; (**b**) 5; (**c**) 10; (**d**) 15; and (**e**) 20 mass. p.

Microscopic studies of the phase structure of the cured compositions are shown in Figure 12. The SEM images show that the introduction of the modifier leads to the formation of a heterogeneous structure, which confirms the absence of complete incorporation of m-EVA40 into the chemical network with tBA. That is, during photopolymerization, accompanied by an increase in the molecular weight of tBA in a mixture with EVA, there is an increase in viscosity and a decrease in the thermodynamic compatibility of the components, leading to the formation of heterogeneous phase structures in the system. It is evident that the introduction of already 5 mass. p. of the modifier leads to the formation of a structure of the “interpenetrating phases” type, and an increase in the concentration to 10 mass. p. changes the phase organization towards “inverted matrix-dispersion”, where the dispersed phase is enriched with tBA. The conclusion about the phase composition is made on the basis of data on the volume fraction of the dispersed phase and the diffusion mobility of the initial components of the system [24]. A further increase in the concentration of the components leads to a decrease in the size of the phase particles, which is associated with the onset of phase decomposition at a later stage of the photochemical polymerization reaction [40].

Thus, studies of the phase structure of the photopolymerized tBA–m-EVA40 system confirm the data presented above. The introduction of m-EVA40 into tBA does not lead to the formation of a spatial network of chemical bonds, i.e., the copolymer does not act as a full-fledged crosslinking agent, but is a thermoplastic modifier and thickener, affecting the operational and technological properties of the material, respectively.

The fundamental focus of the work on studying the possibility of the complex use of the modifying heat-treated additive m-EVA did not include studying the operational properties of photosensitive modified systems, but was determined by the establishment of specific functional interactions between the components during the curing of such systems. Thus, in continuation of this work, a crosslinking agent will be selected, and phase equilibria and structure formation will be investigated during the chemical reaction of photocuring of a tBA-based system modified by EVA, with the study of the influence of the phase structure on the physical and mechanical characteristics of the additive material.

## 4. Conclusions

This work tested the possibility of using EVA40 in LPSCs based on acrylates as a complex additive capable of not only being a thermoplastic modifier that improves physical and mechanical properties at low temperatures, but also acting as a thickener that improves the processability of systems, and also acting as an additional crosslinking agent due to the appearance of C=C double bonds after heat treatment.

The results obtained by FTIR spectroscopy, liquid NMR spectroscopy, and spectrophotometry showed that short-term heat treatment of EVA40 allows the formation of a certain amount of C=C bonds in place of VA monomer units in macromolecules without complete decomposition of the original chemical structure of EVA.

The study of the prepared MPSCs based on tBA containing from 0 to 20 mass. p. of m-EVA40 made it possible to trace its effect on the rheological properties in the initial uncured state, the photopolymerization process upon irradiation, and structure formation during photocuring. The addition of thermoplastic to the photosensitive composition in an amount of 10 mass. p. and higher leads to a sharp increase in the dynamic viscosity and a change in the flow pattern from Newtonian to pseudoplastic in the shear rate range of 0.1–100 s^−1^. The introduction of even 5 mass. p. of m-EVA40 into the system slows down the rate of the photochemical polymerization reaction and reduces the degree of MPSC conversion, despite the presence of C=C bonds in the thermoplastic. At the maximum m-EVA40 content of 20 mass. p., MPSC is capable of stably polymerizing upon irradiation with the consumption of C=C double bonds, which is confirmed by the FTIR method. The heterogeneous phase structure of all modified cured systems confirms that m-EVA40 is not a crosslinking agent, but works as a thermoplastic modifier. The absence of a spatial network of chemical bonds is confirmed by 24 h exposure to solvents at room temperature.

Thus, when introducing m-EVA40 up to 20 mass. p. into a model photosensitive composition based on tBA, despite the presence of C=C double bonds in its molecular structure, the thermoplastic acts as a thickener and modifier. In real systems modified with m-EVA40, in order to form a dense network of chemical bonds during photocuring, it is necessary to use another oligomer or crosslinking agent in a pair with tBA, which is planned to be performed at the next stage of the study.

## Figures and Tables

**Table 1:** Physicochemical properties of the initial components.

No.	Abbreviation	*ρ*, [g/cm^3^]	*n_D_*^20^, [a.u.]	*Т_g,inf_*, [°C]	*T_m,p_*, [°C]	*X_c_*, [%]	*M_n_*, [Da]	*M_w_*, [Da]
a	tBA	0.883 ^1^	1.4119 ^2^	–	−69.0 ^1^	–	128.17 ^1^	128.17 ^1^
b	BAPO	1.170 ^1^	1.5880 ^1^	–	132.9 ^1^	–	418.46 ^1^	418.46 ^1^
c	EVA40	0.959 ^1^	1.4754 ^2^	−28.6 ^3^	47.1 ^3^	15.39 ^3^	48,452.0 ^4^	124,748.0 ^4^

*ρ*—density at 20 °С; n*_D_*^20^—refractive index at 20 °C and wavelength 589.3 nm; *Т_g,inf_*—glass transition temperature (temperature at the inflection point of the S-shaped change in heat capacity on the DSC thermogram); *T_m,p_*—melting temperature (temperature corresponding to the maximum of the endothermic peak on the DSC thermogram); *Χ_c_*—degree of crystallinity according to DSC [13]; M_n_—number-average molecular weight; *M_w_*—weight-average molecular weight. ^1^ Provided by the manufacturer. ^2^ According to refractometry in direct transmission mode—ATAGO NAR-2T (Atago Co., Ltd., Tokyo, Japan). ^3^ Obtained using Differential Scanning Calorimetry (DSC) after the second heating—NETZSCH DSC 204 F1 Phoenix (Netzsch-Geratebau GmbH, Selb, Germany). ^4^ Obtained using Gel Permeation Chromatography (GPC) at 25 °C relative to linear polystyrene—Waters Breeze 2 SPA with Waters Styragel HR 4E and 5E columns (Waters Corp., Milford, MA, USA).

**Table 2:** The composition of the studied MPSCs.

Abbreviation of MPSC	tBA, [mass. p.]	m-EVA40, [mass. p.]	BAPO, [mass. p.]	tBA, [wt.%]	m-EVA40, [wt.%]	BAPO, [wt.%]
0 EVA	100	0	1	99.0	0	1.0
5 EVA	100	5	1	94.34	4.72	0.94
10 EVA	100	10	1	90.09	9.01	0.90
15 EVA	100	15	1	86.20	12.94	0.86
20 EVA	100	20	1	82.64	16.53	0.83

**Table 3:** Rheological properties of MPSCs.

Abbreviation of MPSC	*τ*_0.1_, [mPa] ^1^	*η*_0.1_, [mPa∙s] ^1^		*n* ^3^	*k*, [Pa∙s^n^] ^3^
		*Mean* ^2^	*SD* ^2^		
0 EVA	0.116	1.15	0.048	1.072	*η*
5 EVA	1.07	10.66	0.27	1.061	*η*
10 EVA	120.02	1200.2	2.71	0.728	*τ*/*γ*^0.728^
15 EVA	438.0	4379.0	315.46	0.722	*τ*/*γ*^0.722^
20 EVA	977.0	9761.0	700.47	0.727	*τ*/*γ*^0.727^

^1^ for Newtonian fluids, the values are obtained by approximating the flow curve with a straight line in the shear rate range of 0.1–100 s^−1^; for pseudoplastic fluids, the values are given for $\dot{\gamma }$ = 0.1 s^−1^. ^2^ *Mean* is the arithmetic mean of a value in a sample of three experiments. *SD* is the standard deviation of a value in a sample of three experiments. ^3^ for Newtonian fluids, the power law is transformed into Newton’s law; the parameter values for a pseudoplastic fluid are given by approximating the flow curve (in logarithmic scales) with a straight line in the region with a viscosity anomaly [30]. SD is the standard deviation of η_0.1_ in a sample of three experiments.

**Table 4:** Results of the analysis of experimental data of the Photo-DSC method for MPSCs.

Abbreviation of MPSC	*t_peak_*, [s] ^1^	*Q_exp_,* [J/g] ^2^	*Q_theor_,* [J/g] ^3^	*τ_инд_*, [s] ^4^	*α,* [%] ^5^
0 EVA	134	570.90	625.0	84	91.34
5 EVA	138	563.08	625.0	85	90.09
10 EVA	141	560.63	625.0	81	89.70
15 EVA	144	563.97	625.0	77	90.23
20 EVA	143	549.19	625.0	80	87.87

^1^ obtained by analyzing Figure 8a. ^2^ calculated using Equation (1). ^3^ calculated using Equation (5). ^4^ obtained by analyzing Figure 8b. ^5^ calculated using Equation (4).

## Data Availability

The original contributions presented in this study are included in the article/Supplementary Material. Further inquiries can be directed to the corresponding author.

## Supplementary Materials

The following supporting information can be downloaded at: https://www.mdpi.com/article/10.3390/polym17202787/s1, Figure S1: Photo of the design of the ABS plastic cover for the DSC furnace; Figure S2: Schematic diagram of the developed Photo-DSC device; Figure S3: Photo of NIR filter for halogen lamp OSL2B; Figure S4: Continuous emission spectra at 25 °C of the OSL2B lamp, recorded at four power factors through an NIR filter; Figure S5: Characteristics of silicate glasses used in the Photo-DSC method: (a) X-ray spectrum; (b) continuous absorption spectrum (at 25 °C); Table S1: Results of quantitative analysis of a glass sample used in the Photo-DSC method.
