# Supplementary material for: Ethylene-Vinyl Acetate Copolymer as a Polyfunctional Modifier for Low-Viscosity Photosensitive Compositions
Source: Polymers (Basel). 2025 Oct 17;17(20):2787. doi: 10.3390/polym17202787 (PMC12566735; doi:10.3390/polym17202787)
Supplement: Supplementary file 1 [file polymers-17-02787-s001.zip › polymers-3897119-supplementary.pdf]

SUPPLEMENTARY MATERIALS

Ethylene-Vinyl Acetate Copolymer as a Polyfunctional Modifier for Low-Viscosity Photosensitive Compositions

Dmitriy A. Bazhanov, Uliana V. Nikulova, Ramil R. Khasbiullin, Nikita Yu. Budylin, Elizaveta V. Ermakova and Aleksey V. Shapagin[*]

Frumkin Institute of Physical Chemistry and Electrochemistry Russian Academy of Sciences (IPCE RAS)
E-mail: shapagin@mail.ru

Modification of the design of a conventional differential scanning calorimeter (DSC) to convert it into a differential scanning photocalorimeter (Photo-DSC)

The design of the conventional NETZSCH DSC 204 F1 Phoenix device (Netzsch-Geratebau GmbH, Selb, Germany) was modified by replacing the standard silver block cover with a specially made part made of acrylonitrile butadiene styrene (ABS plastic). The cover we made has two holes with an outer diameter of ∅14.5 mm and an inner diameter of ∅8.5 mm for attaching double flexible fiber light guides. The larger diameter hole is needed to insert the optical waveguide tip, and the smaller one is for the passage of radiation through the block. A photo of the design of the cover we made is shown in **Figure S1**.

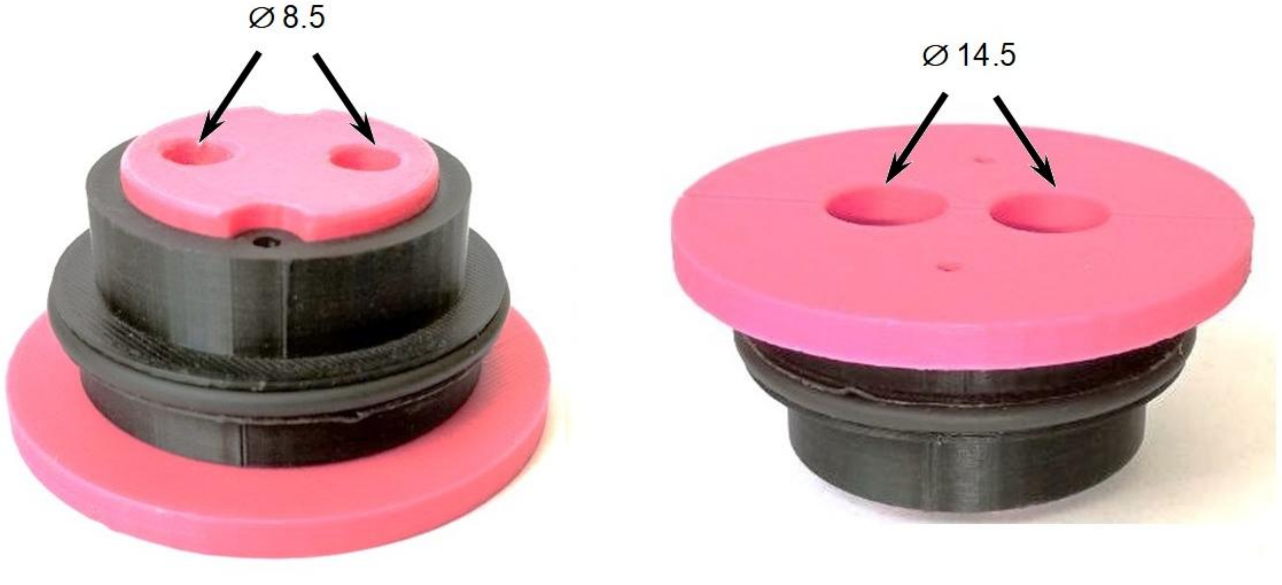

Figure S1. Photo of the design of the ABS plastic cover for the DSC furnace.

The resulting cover made it possible to connect a Thorlabs OSL2 Fiber Illuminator prefix-light source (Thorlabs Inc., Newton, NJ, USA) to the DSC furnace and to place double flexible fiber light

guides over aluminum crucibles. In the resulting Photo-DSC device, one fiber light guide is focused directly on the crucible with the photosensitive composition sample, and the second is focused on the reference crucible. The distance from the fiber light guide to the crucible is 13 mm. The schematic diagram of the developed Photo-DSC device is shown in **Figure S2**.

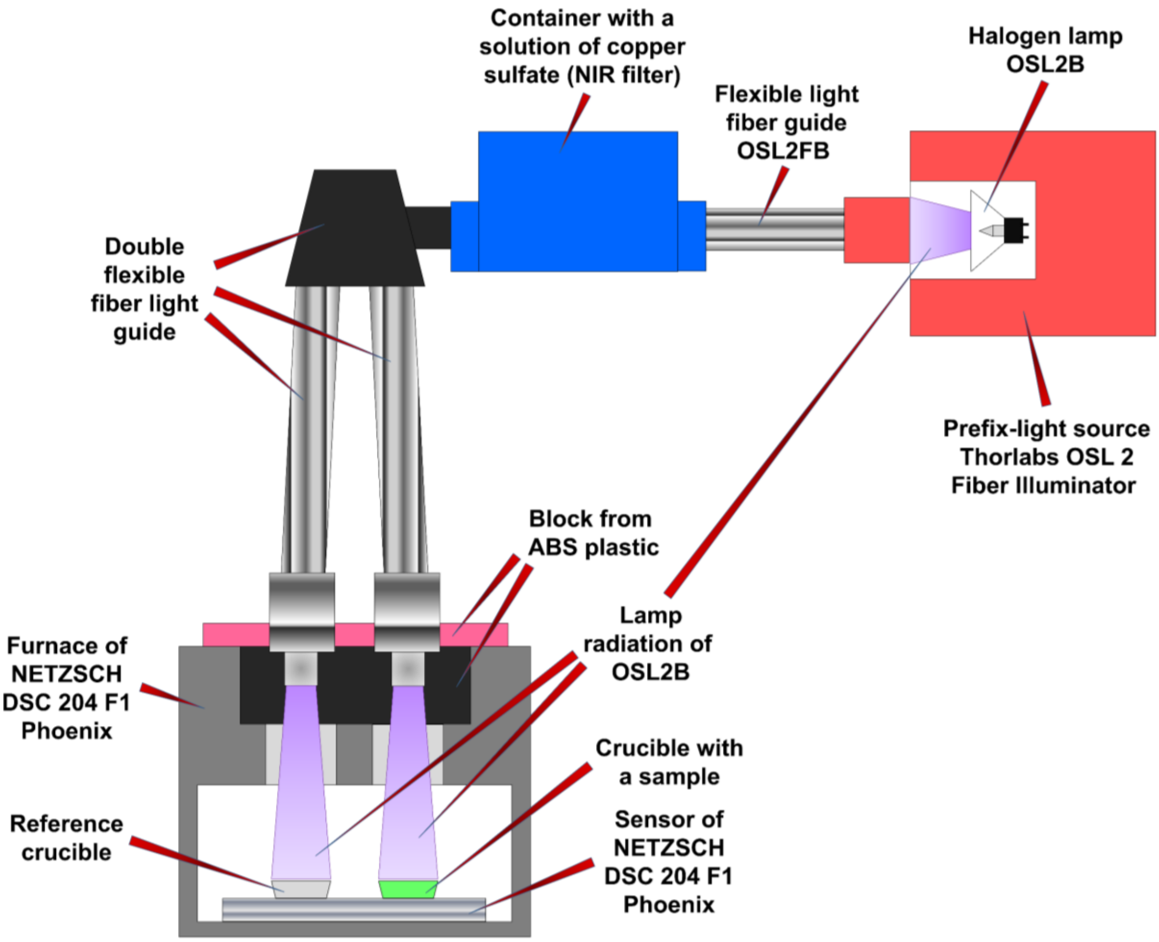

Figure S2. Schematic diagram of the developed Photo-DSC device.

The radiation source of the Photo-DSC prefix-light source is a high-output halogen lamp, OSL2B (Thorlabs Inc., Newton, NJ, USA) of 150 W. In the manual for the prefix-light source, the manufacturer states the operating wavelength range of the lamp as 400–1100 nm. Accordingly, a special filter must be used to absorb the near-infrared radiation (NIR) of the lamp during the experiment. In our study, we used a container with a 0.1 M copper sulfate solution as the NIR filter. A photo of the used NIR filter is shown in **Figure S3**. Continuous emission spectra of the lamp with an NIR filter at four power factors (PF) (**Figure S4**) were measured in air at a room temperature of ~25°C using a highly sensitive fiber optic spectrophotometer Avantes AvaSpec-ULS2048CL-EVO (Avantes B.V., Apeldoorn, The Netherlands) equipped with a balanced deuterium halogen light source AvaLight-DH-S (Avantes B.V., Apeldoorn, The Netherlands). Scanning was performed in transmission mode in the spectral range of 200–1100 nm.

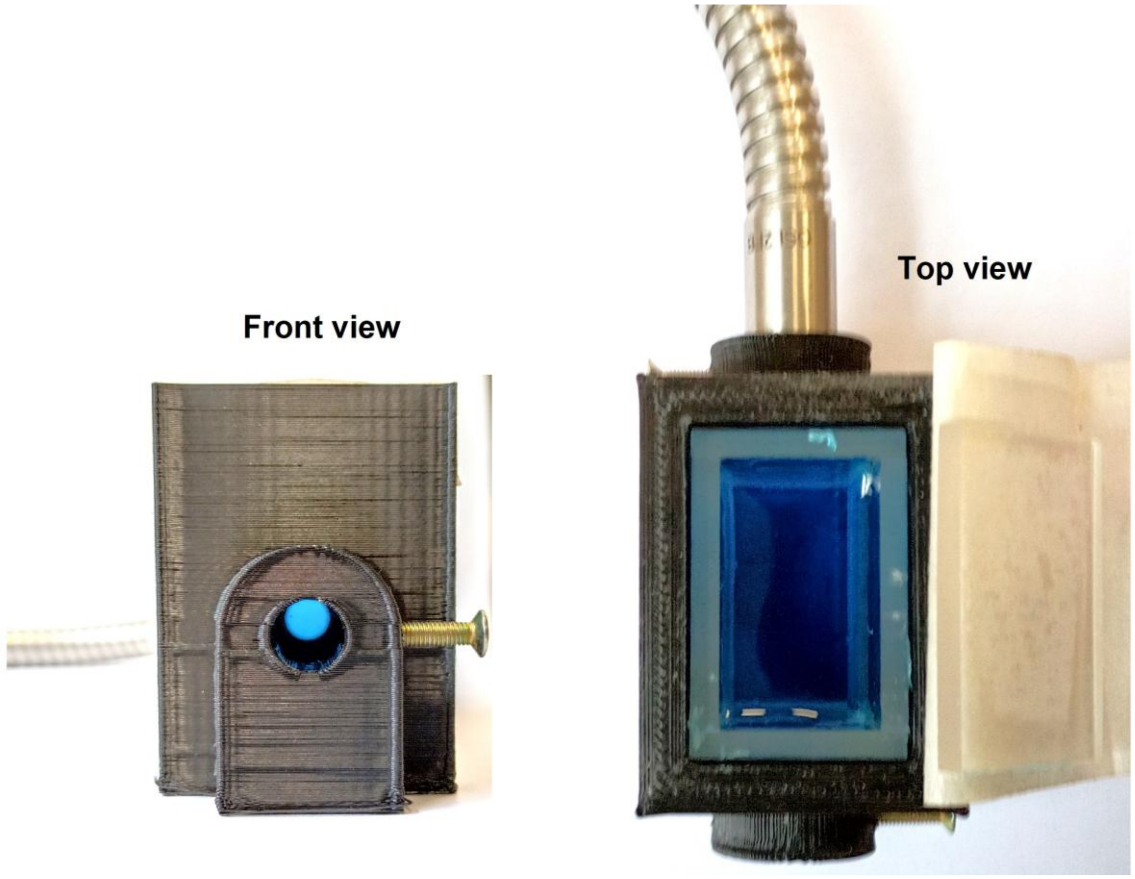

Figure S3. Photo of NIR filter for halogen lamp OSL2B.

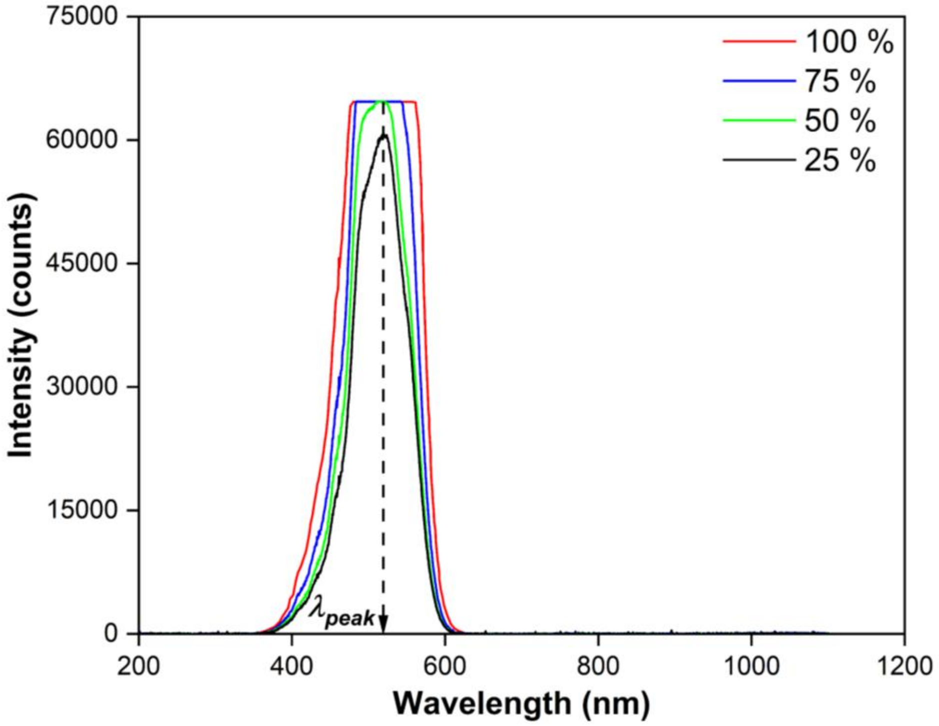

Figure S4. Continuous emission spectra at 25°C of the OSL2B lamp, recorded at four power factors through an NIR filter.

Figure S4 shows that the NIR of the OSL2B lamp in the Photo-DSC prefix-light source is cut off by the filter and thus is not recorded by the spectrophotometer. At the same time, all recorded

emission spectra of the OSL2B lamp with the NIR filter cover the UVA range from 365 to 400 nm. Due to exceeding the maximum measurement limit of the spectrophotometer (64000 units), the peaks of the emission spectra at PF = 75% and 100% are cut off. However, according to the emission spectra at PF = 25% and 50%, it is clear that the peak emission intensity (λ_{peak}) of the OSL2B lamp with the NIR filter falls at 519.2 nm.

To minimize the loss of volatile components of the photosensitive composition (in our case, the tert-butyl acrylate monomer) during the experiment on the Photo-DSC instrument, open aluminum crucibles were used, which were covered from above with round silicate glasses ∅8 × 0.18 mm (Rhuxyol Co., Ltd., China). These glasses were preliminarily characterized by electron probe X-ray microanalysis on a scanning electron microscope JEOL JSM-U3 (JEOL, Ltd., Akishima, Tokyo, Japan) and by absorption spectral analysis on a spectrophotometer SHIMADZU UV-2450 (Shimadzu Scientific Instruments Inc., Columbia, MD, USA). The X-ray spectrum of the glass sample (**Figure S5a**) was recorded on a graphite substrate at an accelerating voltage of 15 kV, and the continuous absorption spectrum (**Figure S5b**) was recorded in the spectral range from 190 to 900 nm in air at a room temperature of ~25°C.

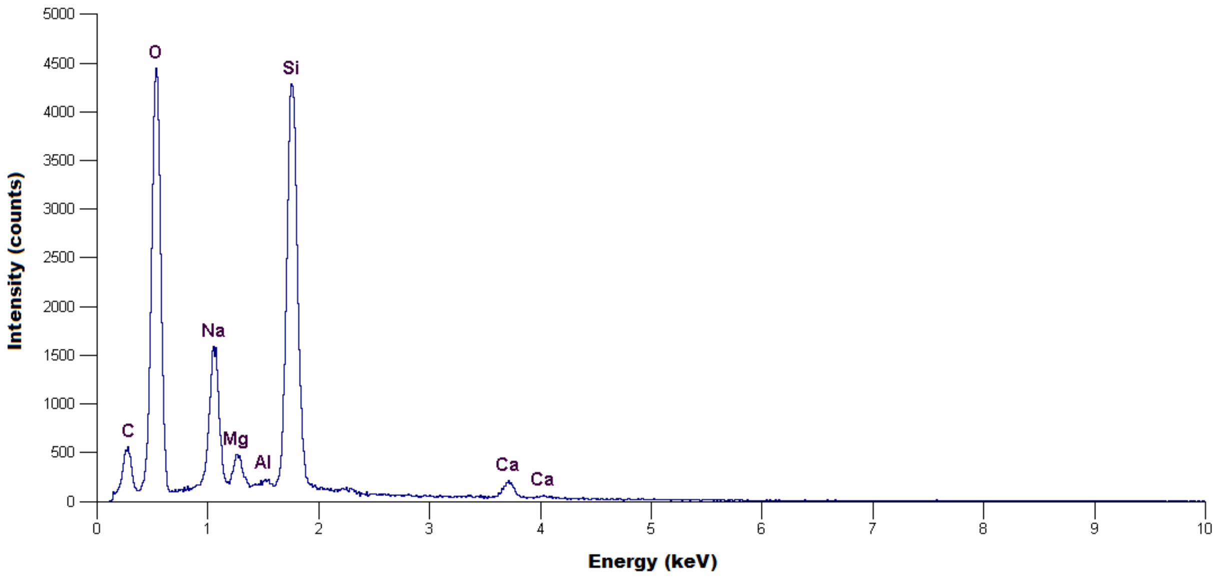

(a)

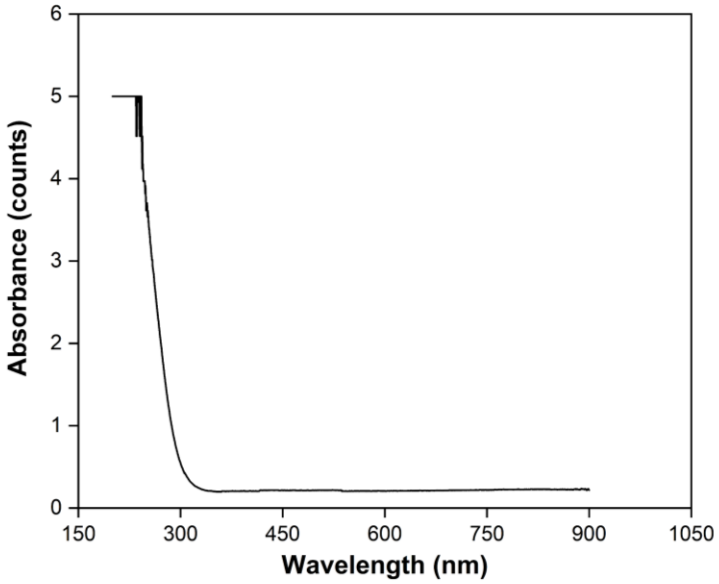

(b)

Figure S5. Characteristics of silicate glasses used in the Photo-DSC method: (a) X-ray spectrum; (b) continuous absorption spectrum (at 25°C).

By means of quantitative analysis operations used in electron probe X-ray microanalysis, the elemental composition of the glass sample was determined (**Table S1**).

Table S1. Results of quantitative analysis of a glass sample used in the Photo-DSC method.

Element	Quantity, [wt.%]	Oxide	Quantity, [wt.%]
Na	12.66	Na_2O	19.18
Mg	2.95	MgO	6.05
Al	0.38	Al_2O_3	0.91
Si	29.16	SiO_2	69.84
Ca	2.25	CaO	4.01
O	52.59	–	–

It is evident from **Figure S5a** and **Table S1** that the glass sample contains mainly silicon (IV) and sodium oxides, as well as a small amount of calcium oxide. This confirms that the glass is silicate and belongs to the sodium-calcium type. At the same time, as can be seen from **Figure S5b**, the glass sample is able to transmit most (~95%) of the radiation in the range of 350–900 nm. The latter fact allows using these glasses in Photo-DSC experiments, since they will transmit the entire spectral range of the lamp radiation, including the one initiating the photochemical reaction of polymerization of modeling photosensitive compositions (MPSCs) based on tBA.
